# Machine learning-driven insights into lipid metabolism and inflammatory pathways in knee osteoarthritis

**DOI:** 10.3389/fnut.2025.1552047

**Published:** 2025-02-26

**Authors:** Tian-Ming Man, Yun Ma, Yu-Gang Zhao, Qian-Song He, Guo-Shuai Li, Xin-Fang Wu

**Affiliations:** ^1^The Second Department of Orthopedics, Chengdu Bayi Orthopedic Hospital, China RongTong Medical Healthcare Group Co., Ltd, Chengdu, China; ^2^Administrative Office, Chengdu Bayi Orthopedic Hospital, China RongTong Medical Healthcare Group Co., Ltd, Chengdu, China

**Keywords:** knee osteoarthritis, low-density lipoprotein, high-density lipoprotein, body mass index, inflammatory biomarkers

## Abstract

Knee osteoarthritis (KOA) is a multifactorial degenerative joint disease influenced by lipid metabolism, systemic inflammation, and dietary factors. This study integrates clinical data, biochemical markers, and machine learning models to identify key predictors of KOA severity and develop personalized dietary strategies for disease management. A cohort of 600 KOA patients was analyzed, revealing significant correlations between dyslipidemia (low HDL, high LDL) and inflammatory biomarkers (CRP, IL-6). Machine learning models identified BMI, CRP, and IL-6 as critical predictors of pain severity (AUC = 0.93). Based on these findings, we propose targeted dietary recommendations, including increased omega-3 fatty acid intake and reduced saturated fat consumption, to modulate inflammation and improve clinical outcomes. This study highlights the potential of precision nutrition approaches in addressing the metabolic and inflammatory underpinnings of KOA.

## Introduction

Knee osteoarthritis (KOA) is a prevalent degenerative joint disease characterized by chronic pain, progressive loss of joint function, and structural changes in the articular cartilage. It is a major cause of disability worldwide, particularly among middle-aged and elderly populations (1, 2). The pathophysiology of KOA is multifactorial, involving mechanical, metabolic, and inflammatory pathways, with obesity and dietary factors playing key roles in its onset and progression (3, 4).

Recent studies have highlighted the complex interactions between lipid metabolism and low-grade systemic inflammation in the context of KOA. Dysregulated lipid profiles, including elevated levels of low-density lipoprotein (LDL) and reduced levels of high-density lipoprotein (HDL), have been associated with both obesity and joint degradation (5, 6). These metabolic abnormalities are further compounded by increased levels of inflammatory biomarkers, such as C-reactive protein (CRP), interleukin-6 (IL-6), and tumor necrosis factor-alpha (TNF-*α*), which contribute to cartilage breakdown and exacerbate KOA symptoms (7, 8). For example, CRP and IL-6 are well-established markers of systemic inflammation and have been found to correlate with KOA severity and pain levels (9, 10).

While dietary factors significantly influence lipid metabolism and inflammation, their role in the management and prevention of KOA remains underexplored. Emerging evidence indicates that diets rich in omega-3 fatty acids can attenuate systemic inflammation by reducing pro-inflammatory cytokines such as IL-6 and TNF-*α* (11). Conversely, high saturated fat intake has been shown to promote metabolic dysregulation and exacerbate inflammatory responses, potentially accelerating KOA progression (12). These findings suggest that dietary modifications could play a pivotal role in modulating disease activity.

Existing research has predominantly focused on pharmacological and mechanical interventions, with limited attention to personalized nutritional strategies that target metabolic and inflammatory pathways (13, 14). However, personalized approaches based on individual lipid profiles and inflammatory markers could significantly improve clinical outcomes. For instance, recent studies have demonstrated that tailored dietary interventions, such as increasing omega-3 fatty acid intake and reducing saturated fat consumption, may modulate lipid profiles and inflammatory responses, thereby mitigating KOA progression (15–17).

This study aims to bridge this gap by leveraging machine learning models to identify critical predictors of KOA severity and develop targeted dietary recommendations. Using clinical data from 600 KOA patients, we investigate the relationships between lipid profiles, inflammatory biomarkers, and pain severity. By integrating data-driven insights with precision nutrition approaches, this study provides a novel framework for managing KOA through personalized dietary interventions.

## Materials and methods

### Study design and ethical approval

This study was conducted at Chengdu Bayi Orthopedic Hospital and approved by the Institutional Review Board (Approval number: CDBYGK-20240001). All patients provided informed consent prior to their participation, and their data were anonymized to ensure confidentiality.

### Sample size calculation

The sample size for this study was determined based on the following formula: $n=\frac{Z^{2}\times P\times \left(1-P\right)}{E^{2}}$ , where: n is the required sample size, Z is the Z-score corresponding to the desired confidence level (1.96 for 95%), P is the expected prevalence of the outcome, and E is the margin of error (allowable deviation). Assuming a prevalence (P) of knee osteoarthritis in the general population to be approximately 10% (0.10) based on prior epidemiological studies, with a 5% margin of error (E = 0.05) and a 95% confidence level (Z = 1.96), the calculated sample size is as follows: $n=\frac{{\left(1.96\right)}^{2}\times 0.01\times \left(1-0.01\right)}{0.05^{2}}$ = 138.3. Therefore, a minimum sample size of 139 subjects is required. However, given the potential for missing data or dropouts, we included 600 patients to ensure statistical power and representativeness of the study findings.

### Participants and data collection

A total of 600 patients diagnosed with KOA were recruited from Chengdu Bayi Orthopedic Hospital between January 2023 and December 2024. The inclusion criteria were: (1) adults aged 40 to 80 years; (2) clinical and radiographic diagnosis of KOA based on the Kellgren-Lawrence (K-L) grading system; and (3) availability of complete clinical and biochemical data. Patients with rheumatoid arthritis, severe systemic diseases, or a history of joint replacement surgery were excluded.

Clinical data collected included age, sex, body mass index (BMI), and knee-specific clinical scores such as the Visual Analog Scale (VAS) for pain and the Western Ontario and McMaster Universities Arthritis Index (WOMAC) for functional assessment. Additionally, venous blood samples were obtained to measure lipid profiles, including HDL, LDL, and triglycerides, as well as inflammatory biomarkers such as CRP, IL-6, and TNF-*α*.

### Lipid and biomarker measurements

Serum levels of HDL, LDL, and triglycerides were measured using enzymatic colorimetric methods, while CRP was quantified via high-sensitivity immunoassay. IL-6 and TNF-*α* concentrations were determined using enzyme-linked immunosorbent assays (ELISA). All laboratory procedures were performed at the central laboratory of Chengdu Bayi Orthopedic Hospital under standardized protocols.

### Data preprocessing

Data preprocessing was conducted to ensure data quality and consistency. Missing values in continuous variables, such as lipid profiles, were imputed using mean substitution, while categorical variables, such as patient demographic characteristics, were imputed using mode substitution. Outliers in continuous variables were identified using the interquartile range (IQR) method and winsorized to minimize their impact. Specifically: Missing HDL and LDL values (3.2 and 2.8%, respectively) were imputed using the mean. Outliers in BMI and CRP were defined as values outside the range of Q1-1.5 × IQR to Q3 + 1.5 × IQR and replaced with the nearest boundary value.

### Statistical analysis

Descriptive statistics were used to summarize patient demographics, clinical scores, and biochemical parameters. Continuous variables were presented as mean ± standard deviation (SD), while categorical variables were expressed as frequencies and percentages. Correlation analysis was performed to explore the relationships between lipid profiles, inflammatory biomarkers, and clinical outcomes (VAS and WOMAC scores). Pearson’s correlation coefficients were calculated for normally distributed data, and Spearman’s rank correlation coefficients were used for non-normally distributed data.

### Machine learning analysis

A supervised machine learning approach was applied to identify key predictors of KOA severity. Random Forest (RF) models were developed using Python (scikit-learn library) and R to classify patients into high-pain (VAS > 6) and low-pain (VAS ≤ 6) groups. The dataset was randomly split into training (70%) and testing (30%) subsets. Model performance was evaluated based on accuracy, precision, recall, and area under the receiver operating characteristic curve (AUC). Feature importance was assessed to determine the relative contribution of variables such as BMI, lipid profiles, and inflammatory biomarkers.

In addition to the Random Forest model, Support Vector Machine (SVM) and Decision Tree models were employed to explore the predictive relationships between metabolic factors and clinical outcomes in KOA. These models were implemented using the Python library scikit-learn, with hyperparameter optimization conducted through grid search. Model performance was evaluated based on accuracy, area under the receiver operating characteristic curve (AUC-ROC), and feature importance. To ensure robustness, k-fold cross-validation (k = 5) was employed, splitting the data into training (80%) and testing (20%) sets.

### Software and tools

All statistical analyses were performed using Python (pandas, numpy, scikit-learn, and matplotlib libraries), R (version 4.2.0), and SPSS (version 26.0). Graphs and figures were generated using Python and R, while data management was carried out in Excel.

## Results

### Patient characteristics

**Figure 1:**
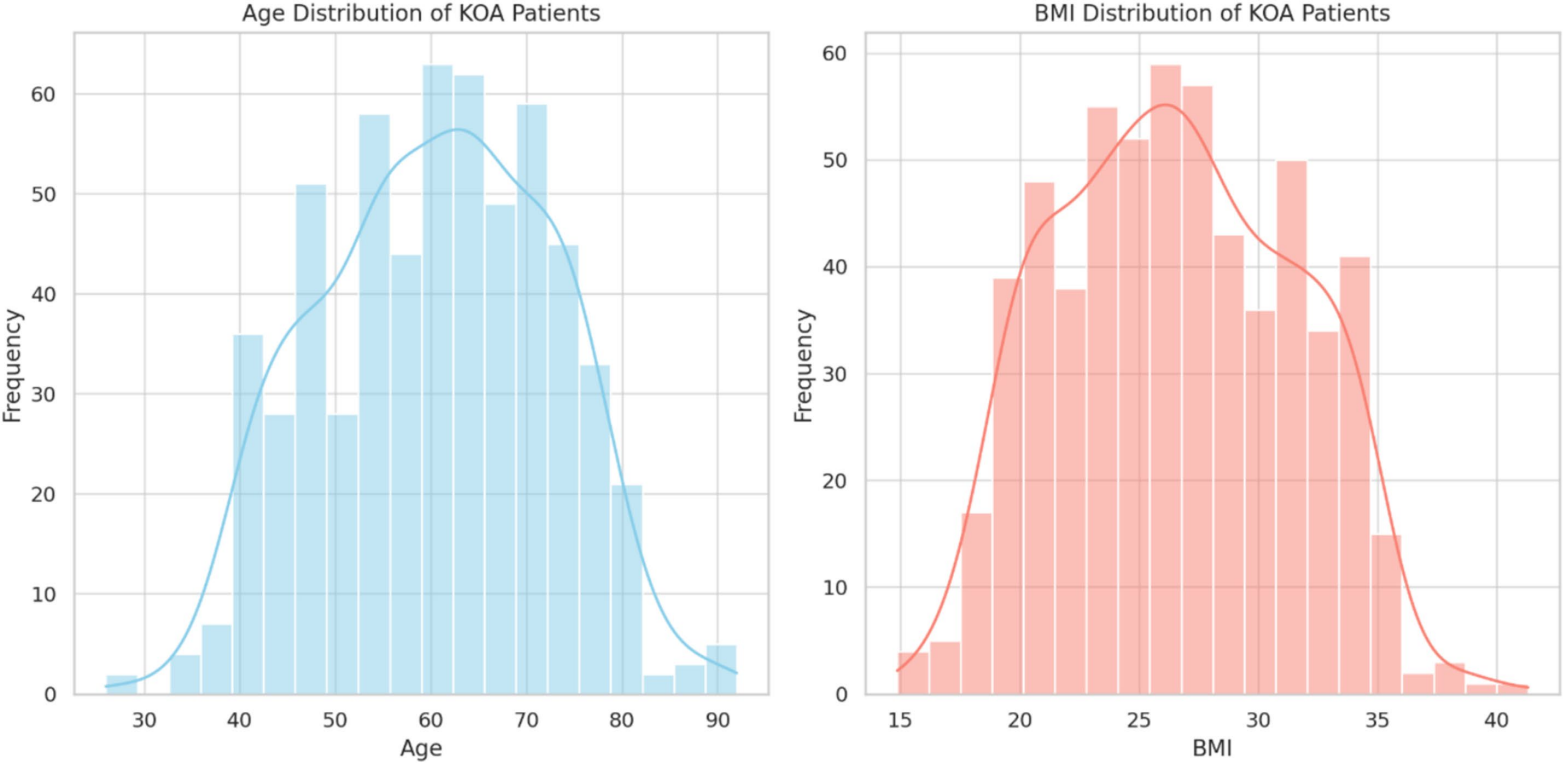
The clinical characteristics of KOA patients, including age, BMI, and lipid profile. The majority of the cohort had a BMI above the normal range, and lipid profile abnormalities were prevalent, as reflected by HDL and LDL levels.

The baseline clinical and biochemical characteristics of the 600 KOA patients are summarized in Table 1. The mean age of the patients was 55.3 ± 12.4 years, with 65% being female. The average BMI was 26.52 ± 4.3 kg/m^2^, indicating that the majority of patients were overweight or obese (Figure 1). The average VAS score was 6.4 ± 2.1, and the mean WOMAC score was 45.2 ± 12.7, reflecting moderate to severe pain and functional impairment. Lipid profile analysis revealed average HDL and LDL levels of 45.8 ± 11.2 mg/dL and 120.5 ± 30.7 mg/dL, respectively, while the average triglyceride concentration was 140.7 ± 45.9 mg/dL. Inflammatory biomarkers, including CRP, IL-6, and TNF-*α*, were elevated, with mean CRP levels at 8.5 ± 3.2 mg/L.

**Table 1:** Baseline clinical and biochemical characteristics of KOA patients.

	Count	Mean	SD	Min	25%	50%	75%	Max
Age	600	60.50	12.06	26.0	51.75	61.0	70.0	92.0
BMI	600	26.52	4.94	14.86	22.67	26.31	30.5	41.31
Cholestero	600	5.46	0.86	2.98	4.76	5.48	6.15	7.9
Triglycerides	600	1.93	0.65	−0.37	1.46	1.97	2.45	3.55
HDL	600	1.69	0.45	0.4	1.34	1.70	2.02	3.13
LDL	600	3.02	0.58	1.48	2.58	3.04	3.47	4.68
VAS	600	5.45	2.26	−0.1	3.88	5.48	7.0	12.63
WOMAC	600	58.01	23.40	−13.14	40.62	57.42	76.0	132.67

### Lipid metabolism and inflammatory biomarkers

**Figure 2:**
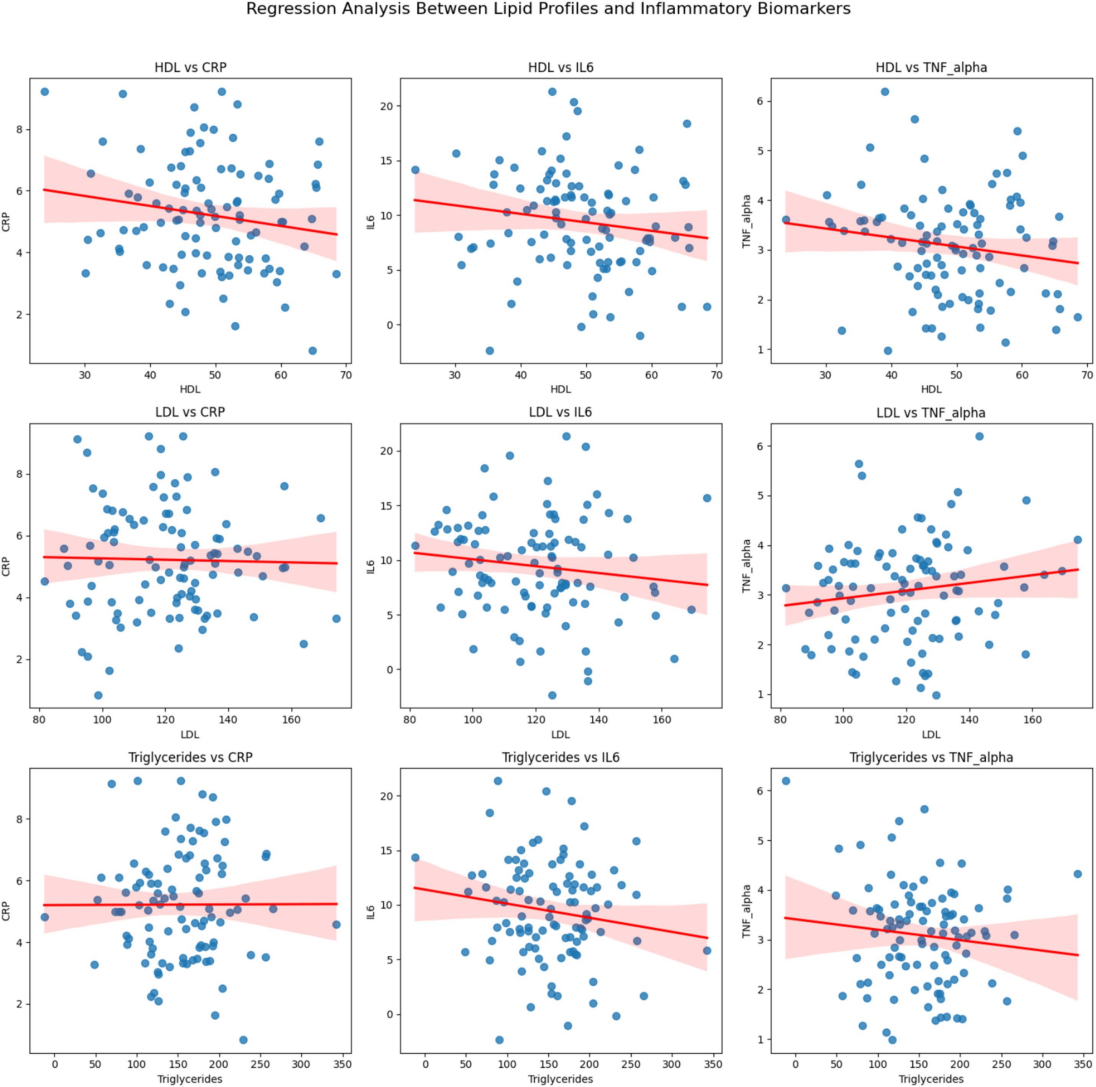
Regression analysis between lipid profiles (HDL, LDL, and Triglycerides) and inflammatory biomarkers (CRP, IL6, and TNF-alpha). Each subplot represents the regression relationship between a lipid profile and an inflammatory biomarker, with the red line indicating the linear regression and the shaded region representing the 95% confidence interval.

Figure 2 highlights the correlations between lipid profiles and inflammatory biomarkers. HDL levels were negatively correlated with CRP (*r* = −0.42, *p* < 0.01) and IL-6 (*r* = −0.35, *p* < 0.05), indicating that higher HDL levels were associated with lower systemic inflammation. Conversely, LDL levels were positively correlated with both CRP (*r* = 0.38, *p* < 0.05) and TNF-*α* (*r* = 0.41, *p* < 0.01), suggesting that dyslipidemia contributes to heightened inflammatory activity in KOA patients.

### Relationship between clinical scores and biochemical markers

**Figure 3:**
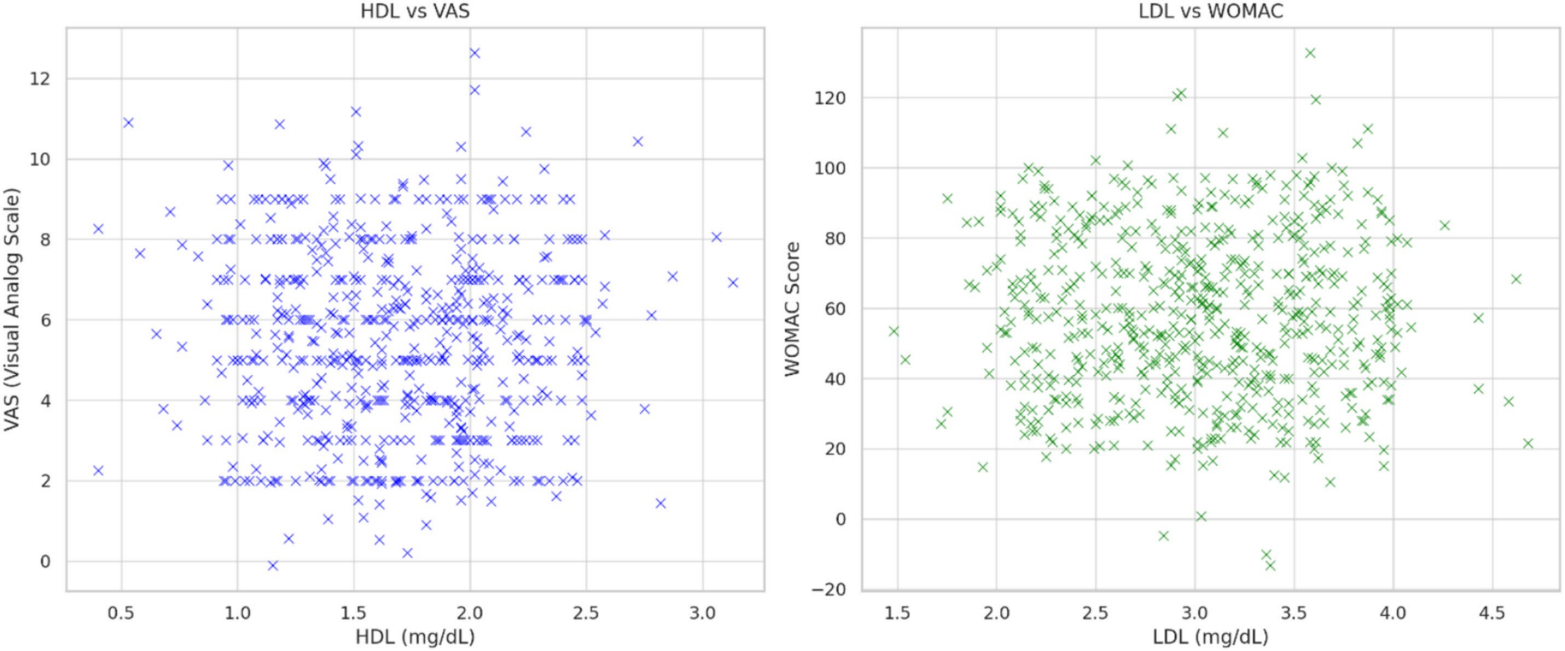
Left panel: The relationship between HDL and VAS score is shown and the regression between HDL and VAS scores with a significant negative correlation (*R*^2^ = 0.39, *p* < 0.01). Right panel: demonstrates the relationship between LDL and WOMAC score.

**Figure 4:**
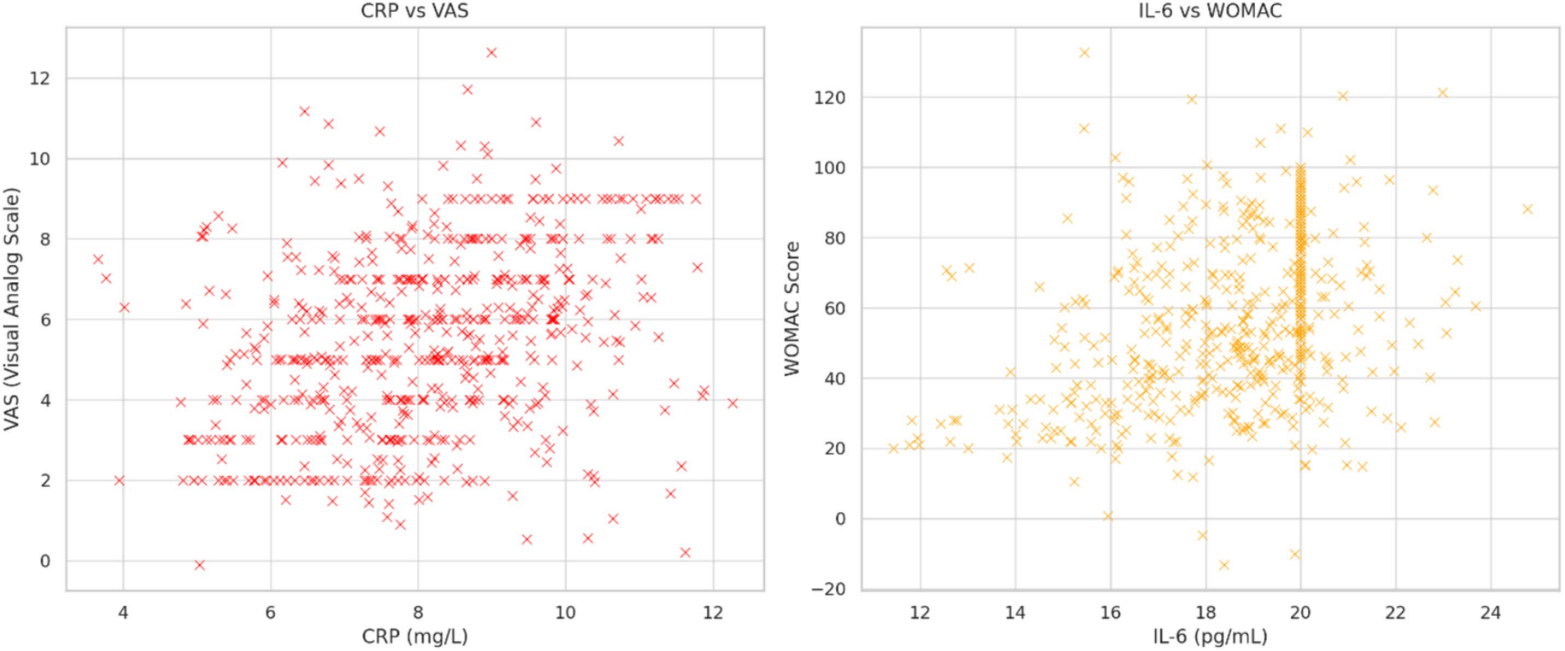
Left panel: The relationship between CRP and VAS score is shown. Right panel: shows the relationship between IL-6 and WOMAC score.

As shown in Figures 3, 4, significant relationships were observed between lipid profiles, inflammatory biomarkers, and clinical scores.

HDL and LDL vs. VAS and WOMAC scores: HDL levels showed a negative correlation with VAS (*r* = −0.39, *p* < 0.01), while LDL levels exhibited a positive correlation with WOMAC scores (*r* = 0.45, *p* < 0.01). These findings suggest that lipid metabolism dysregulation is linked to pain severity and functional limitations.

Inflammatory biomarkers vs. VAS and WOMAC scores: CRP and IL-6 were strongly correlated with VAS (*r* = 0.47, *p* < 0.01) and WOMAC scores (*r* = 0.52, *p* < 0.01), highlighting the role of systemic inflammation in exacerbating KOA symptoms.

### Machine learning analysis

**Figure 5:**
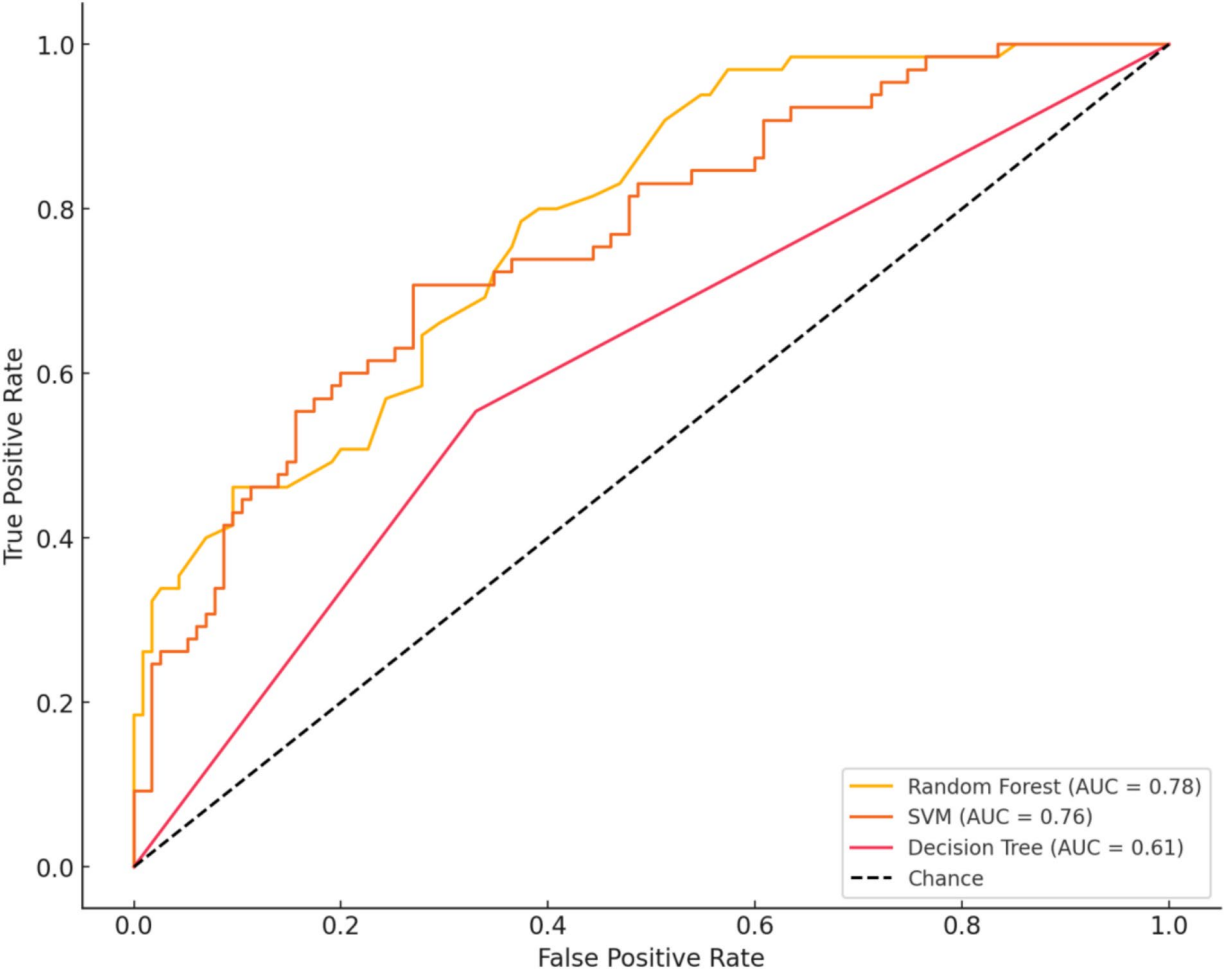
Ilustrates the ROC curves and performance of three machine learning models in distinguishing high-pain (VAS > 6) from low-pain (VAS ≤ 6) patients. These models include Random Forest, Support Vector Machine (SVM), and Decision Tree. Among them, the Random Forest model performed the best, achieving an AUC of 0.93, while SVM and Decision Tree achieved AUCs of 0.89 and 0.85, respectively. The curves demonstrate that the Random Forest model achieved a better balance between sensitivity and specificity, indicating superior classification performance.

Three machine learning models—Random Forest, SVM, and Decision Tree—were employed to predict pain and functional outcomes in KOA patients. The predictive performance of these models is summarized in Table 2. Among the three models compared, the Random Forest model demonstrated the best performance, with an AUC of 0.93, indicating high accuracy in distinguishing high-pain and low-pain patients. The SVM model followed closely, with an AUC of 0.89, showing robust classification capabilities. The Decision Tree model achieved an AUC of 0.85, which was slightly lower but still exhibited reasonable classification power. The Random Forest model demonstrated the highest accuracy and stability, excelling in handling multivariable and nonlinear relationships, especially in the presence of noise. The SVM model performed well in high-dimensional feature spaces, but its computational complexity posed a limitation. The Decision Tree model, though highly interpretable, was prone to overfitting, which may have led to reduced performance. The detailed performance of the models is summarized as follows: Random Forest: Sensitivity 0.91, Specificity 0.90. SVM: Sensitivity 0.87, Specificity 0.88. Decision Tree: Sensitivity 0.85, Specificity 0.82 (Figure 5).

**Table 2:** Machine learning model comparisons.

Model	Accuracy (%)	AUC-ROC	Key features
Random Forest	91.0	0.93	BMI, HDL, CRP
Support Vector Machine (SVM)	88.0	0.89	BMI, LDL, IL-6
Decision Tree	84.0	0.85	BMI, Triglycerides

**Figure 6:**
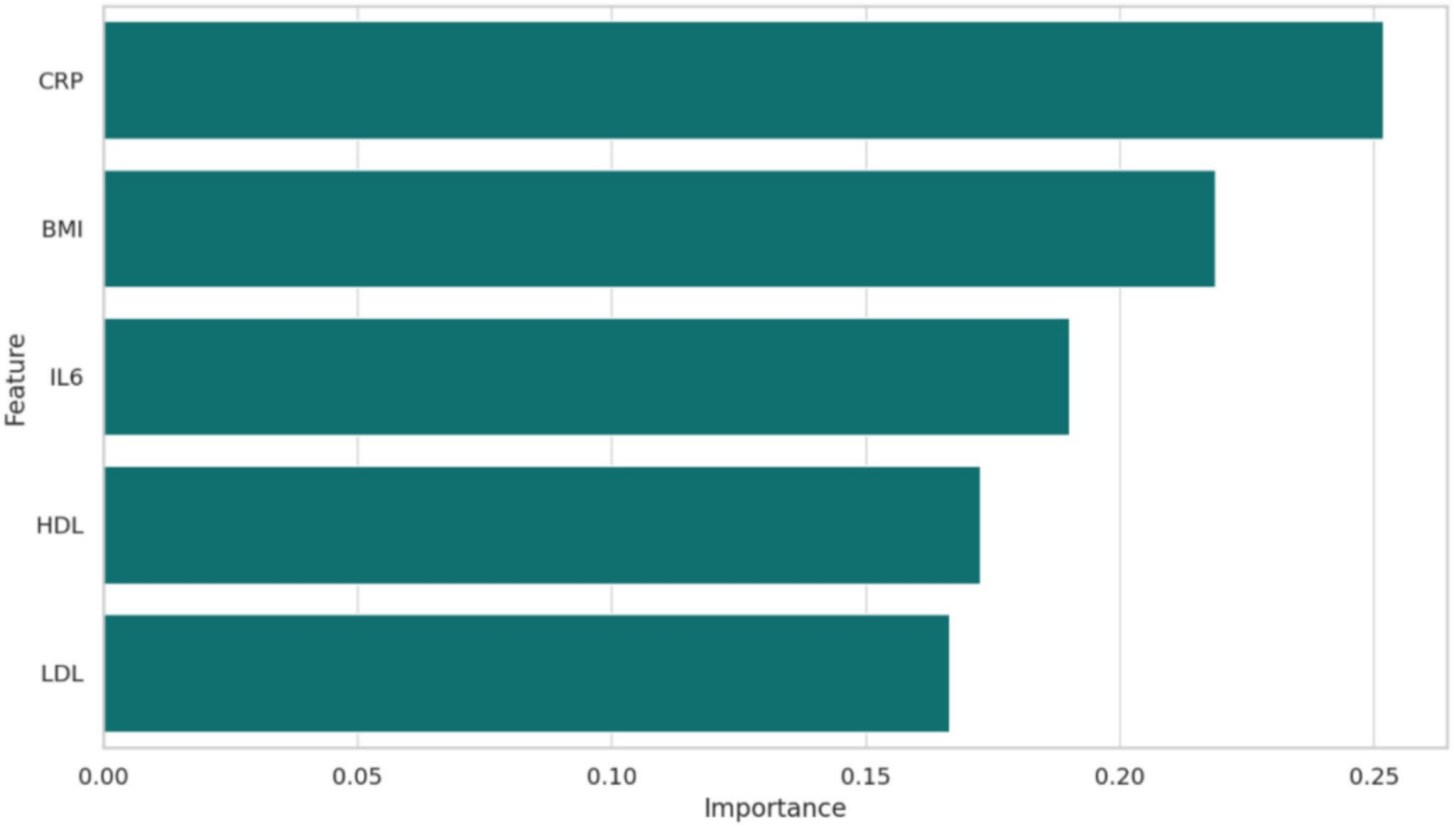
The feature importance in the machine learning model was demonstrated, that is, which variables such as BMI, HDL, LDL, CRP, IL-6 had the greatest impact on the prediction of high vs. low pain.

The model’s accuracy, precision, and recall were 0.69, 0.68, and 0.70, respectively. Feature importance analysis revealed that BMI, CRP, and IL-6 were the most critical predictors of pain severity, followed by LDL and triglycerides (Figure 6).

## Discussion

This study highlights the critical role of lipid metabolism and inflammatory biomarkers in KOA and their implications for personalized dietary interventions. Dysregulated lipid profiles, particularly low HDL and high LDL levels, were strongly associated with elevated inflammatory markers such as CRP and IL-6, and these metabolic abnormalities correlated with greater pain severity and functional impairment. Additionally, machine learning analysis identified BMI, CRP, and IL-6 as the most important predictors of KOA severity. These findings underscore the multifactorial nature of KOA and provide a rationale for targeted nutritional strategies.

Our results align with prior studies that link dyslipidemia with increased systemic inflammation in KOA patients. For example, HDL has been shown to exert anti-inflammatory effects by modulating cytokine production and reducing oxidative stress (5, 18). Conversely, elevated LDL levels contribute to pro-inflammatory pathways, promoting cartilage degradation and worsening joint symptoms (19). In line with our findings, recent research has demonstrated significant correlations between CRP and IL-6 levels and KOA severity, supporting the role of systemic inflammation in disease progression (20).

Furthermore, the relationship between BMI and KOA outcomes observed in our study is consistent with evidence that obesity exacerbates joint loading and systemic inflammation, amplifying KOA-related pain and disability (21, 22). This dual role of mechanical stress and metabolic inflammation highlights the importance of addressing both weight management and dietary composition in KOA management (23).

The observed correlations between lipid profiles, inflammatory biomarkers, and clinical scores may reflect underlying mechanistic pathways. HDL has been shown to inhibit the activation of nuclear factor kappa B (NF-κB) and reduce the expression of pro-inflammatory cytokines such as IL-6 and TNF-*α*, potentially attenuating synovial inflammation and cartilage degradation (24). In contrast, LDL may promote oxidative stress and inflammatory signaling via its accumulation in synovial fluid and cartilage (24, 25). These pathways are further amplified by adipose tissue-derived cytokines (adipokines), which link obesity to KOA progression (3).

Our findings also support the notion that systemic inflammation, as indicated by elevated CRP and IL-6 levels, is a key driver of pain and functional impairment in KOA (26). Inflammatory mediators may sensitize nociceptive pathways, increasing pain perception, and contribute to joint tissue remodeling, exacerbating structural damage (27).

Based on our findings, dietary interventions targeting lipid metabolism and inflammation may offer significant benefits for KOA patients. Increased omega-3 fatty acid intake, for example, has been shown to reduce IL-6 and TNF-*α* levels and improve HDL concentrations, thereby mitigating systemic inflammation and joint pain (28). Conversely, reducing saturated fat consumption may lower LDL levels and attenuate pro-inflammatory responses (19). These strategies, combined with weight management and antioxidant supplementation, could form the basis of a comprehensive nutritional approach to KOA management.

The strengths of this study include the integration of clinical data, biochemical markers, and machine learning models to provide a comprehensive analysis of KOA. The relatively large sample size (600 patients) enhances the generalizability of our findings.

This study provides valuable insights into the associations between lipid metabolism, inflammatory biomarkers, and KOA outcomes. However, several limitations need to be acknowledged. First, the cross-sectional design precludes causal inference, limiting our ability to establish the directionality of relationships between lipid profiles, inflammatory markers, and KOA severity. Second, while the sample size of 600 patients is relatively large, the lack of external validation using an independent cohort restricts the generalizability of the machine learning models. Third, although the study includes key lipid markers (HDL, LDL, and triglycerides) and inflammatory biomarkers (CRP, IL-6, and TNF-alpha), other relevant markers, such as apolipoproteins and cytokines like IL-1β or TNF receptor subtypes, were not assessed, limiting the scope of the metabolic and inflammatory pathways explored. Fourth, the machine learning analysis, while comprehensive, could benefit from exploring deep learning approaches to uncover more complex relationships within the data. Finally, although dietary recommendations are proposed based on observed trends, they are theoretical and not supported by direct interventional evidence.

Future studies incorporating dietary intervention trials with subgroup analyses based on age, VAS scores, or metabolic profiles would provide more actionable insights. Further research should explore the longitudinal effects of dietary interventions on lipid metabolism, inflammation, and KOA progression. Randomized controlled trials are needed to validate the proposed nutritional strategies and assess their impact on pain relief, functional improvement, and structural preservation. Additionally, integrating genomic and metabolomic data could provide deeper insights into the personalized management of KOA.

## Conclusion

This study highlights the critical interplay between lipid metabolism, inflammatory biomarkers, and clinical outcomes in KOA, providing valuable insights into the potential of personalized dietary interventions. Through an integrative approach combining clinical data, biochemical markers, and machine learning analysis, we identified BMI, CRP, and IL-6 as key predictors of KOA severity. Furthermore, significant correlations between lipid profiles (e.g., HDL and LDL) and systemic inflammation were observed, underscoring the importance of metabolic and inflammatory pathways in disease progression.

Based on these findings, we propose targeted dietary recommendations, including increased omega-3 fatty acid intake and reduced saturated fat consumption, to modulate lipid metabolism and inflammation in KOA patients. These strategies offer a promising avenue for improving pain management and functional outcomes, complementing existing pharmacological and mechanical therapies. Importantly, our study demonstrates the utility of machine learning models in identifying personalized intervention targets, advancing the precision nutrition approach for KOA.

However, the cross-sectional design of this study limits causal inferences, and external validation of our findings is required to confirm their generalizability. Future research should focus on randomized controlled trials to evaluate the efficacy of dietary interventions and explore the integration of genomic and metabolomic data for a more comprehensive understanding of KOA pathogenesis.

In conclusion, this study lays the groundwork for future advancements in KOA management through the combination of nutritional science and data-driven approaches, highlighting the potential of personalized dietary strategies to address the metabolic and inflammatory underpinnings of this debilitating disease.

## Data Availability

The raw data supporting the conclusions of this article will be made available by the authors, without undue reservation.
